# Proposal of a Nonlinear Interaction of Person and Situation (NIPS) model

**DOI:** 10.3389/fpsyg.2013.00499

**Published:** 2013-08-02

**Authors:** Manfred Schmitt, Mario Gollwitzer, Anna Baumert, Gabriela Blum, Tobias Gschwendner, Wilhelm Hofmann, Tobias Rothmund

**Affiliations:** ^1^Department of Psychology, University of Koblenz-LandauLandau, Germany; ^2^Department of Psychology, University of MarburgMarburg, Germany; ^3^Family- and Life Counseling, Bishopric TrierTrier, Germany; ^4^Department of Psychology, University of CologneKöln, Germany

**Keywords:** interactionism, person × situation interaction, Rasch model, weak situations, strong situations, weak persons, strong persons

## Abstract

Marshall and Brown (2006) proposed a *Traits as Situational Sensitivities* (*TASS*) *Model*, which implies a systematic person × situation interaction. We review this model and show that it suffers from several limitations. We extend and modify the model in order to obtain a symmetric pattern of levels and effects for both person and situation factors. Our suggestions result in a general Nonlinear Interaction of Person and Situation (NIPS) Model. The NIPS model bears striking similarities to the Rasch model. Based on the symmetric nature of the NIPS model, we generalize the concept of weak and strong situations to individuals and propose the concepts of weak and strong persons. Finally, we discuss psychological mechanisms that might explain the NIPS pattern and offer ideas for future research.

Marshall and Brown (2006) proposed a *Traits as Situational Sensitivities* (*TASS*) model, which addresses an important issue that each theory of behavior has to address: How do personality and situational factors *jointly* shape behavior? The TASS model is a person × situation interaction model. Like all person × situation interaction models, it challenges the assumption that personality and situational factors influence behavior additively. The TASS model assumes a characteristic type of deviation from additivity, and thus a specific person × situation interaction.

Marshall and Brown (2006) use aggression as an example to illustrate their model. The aggression literature has achieved the consensus that aggressive behavior in a given situation is a function of aggression-related personality traits and the potential of the situation to provoke this kind of behavior (cf. Bettencourt et al., 2006). Less agreement has been obtained with regard to *how* the two factors interact to shape behavior. Marshall and Brown (2006) assume a particular form of that interaction: According to their model, situational effects among lower levels of provocation are stronger for highly trait-aggressive individuals, whereas situational effects among higher levels of provocation are stronger for individuals low in trait aggressiveness. Thus, depending on their level of trait aggressiveness, individuals differ in their situational sensitivity; hence, the model's name. Figure 1 displays the original TASS pattern [adopted from Marshall and Brown (2006), Figure 1A]^1^.

**Figure 1:**
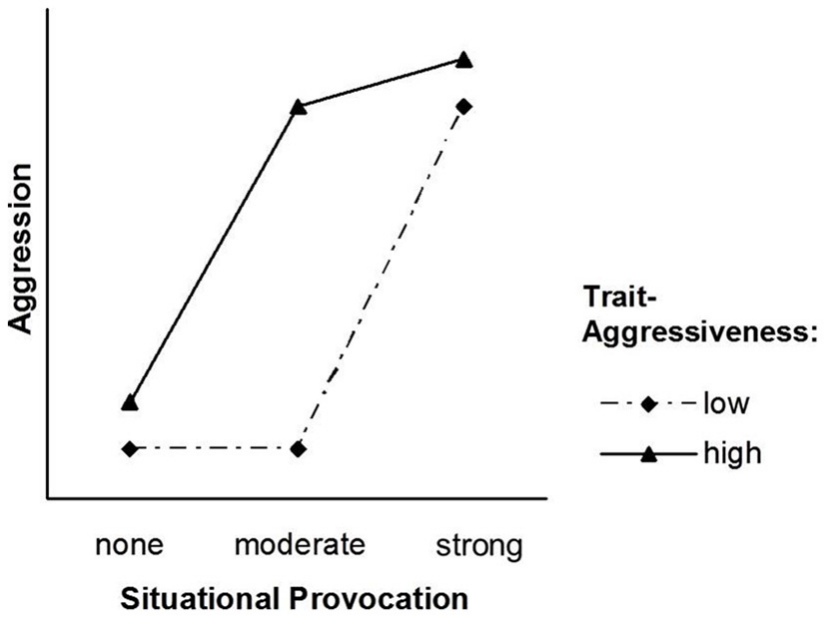
**The original TASS model [adopted from Marshall and Brown (2006), Figure 1A]**.

Although the basic idea of the TASS model is appealing and has provided progress in understanding the joint impact of person and situation factors on behavior, we argue that the particular way it was formulated by Marshall and Brown (2006) rests on three assumptions that are not fully convincing: (a) Whereas situational effects are assumed to be *nonlinear* across trait levels, trait effects are assumed to be *linear* across situation levels. (b) People low in trait aggressiveness are assumed to be unaffected by a moderate provocation. (c) Trait effects are assumed to be virtually zero in situations high in aggression-provocation.

In the first part of this article, we will discuss these assumptions and conclude that they are not sufficiently plausible. In the second part, we will show that the basic idea of the TASS model can be retained by adding a few modifications to it. These modifications led us to propose a general Nonlinear Interaction of Person and Situation (NIPS) model. In the third part of our article, we will introduce the striking similarities between the NIPS model and the Rasch model. By relating the basic ideas of the NIPS model to the psychometric concepts of item difficulty and personality effects, we will propose the concept of weak and strong persons. Finally, in the fourth part, we will address psychological mechanisms that might *explain* the NIPS pattern. We will offer some ideas about such mechanisms.

## Three problematic assumptions of the TASS model

### Are linear trait effects consistent with the core premises of TASS?

Marshall and Brown (2006) specified three levels of the situation factor but only two levels of the person factor. Because an empirical test of nonlinear effects requires at least three levels, the design chosen by Marshall and Brown allows for nonlinear *situation* effects but not for nonlinear *trait* effects. Thus, the authors seem to assume that trait effects are linear. This means that within a given situation containing a particular level of provocation, each increase in trait level yields a constant increase in aggression. Such a linearity assumption, however, is not consistent with the TASS model's interpretation of TASS, and this suggests that trait levels can be conceptualized as individual differences in thresholds for perceiving a situation as a provocation. Highly trait-aggressive individuals have a low threshold for interpreting a situation as a provocation. Moderately trait-aggressive individuals have a moderate threshold, and people low in trait aggressiveness have a high threshold. Whether or not two individuals who differ in trait aggressiveness by a given amount react with a similar or with a different degree of aggression depends on the provocation level of a given situation. If the provocation level is *below* the thresholds of *both* persons, they will react *similarly* (i.e., with no or little aggression). If the provocation level is *above* the threshold of *both* persons, they will also react *similarly* (i.e., with some aggression). However, if the provocation level is *below* the threshold of person A and *above* the threshold of person B, they will react *differently*. Person A will react with no or very little aggression; person B with some or much. Hence, the trait effect cannot be the same across situations that differ in level of provocation. Thus, by implication, linear trait effects are inconsistent with the core idea of TASS.

### Are low trait-aggressive individuals unaffected by a moderate provocation?

The TASS model assumes that the difference between the nonprovoking and the moderately provoking situations is zero for individuals low in trait aggressiveness (see Figure 1). This assumption is not plausible, and it is also not in agreement with Marshall and Brown (2006) own data: In all three studies, low trait-aggressive individuals reported more anger in the moderately provoking situation than in the nonprovoking situation. This difference was significant in Studies 2 (cf. Marshall and Brown, 2006, p. 1106) and 3 (cf. Marshall and Brown, 2006, p. 1108).

### Are trait effects virtually zero in strongly-provoking situations?

A third assumption of the TASS model is that trait effects are very small at both ends of the situational continuum (see Figure 1). Whereas this seems likely for nonprovoking situations, it appears doubtful in strongly provoking situations. Again, Marshall and Brown (2006) data are not consistent with their assumption. High trait-aggressive individuals reported significantly more anger as compared to low trait-aggressive individuals in the strong provocation condition. This difference was significant in all three studies (cf. Marshall and Brown, 2006, p. 1104 for Study 1, p. 1107 for Study 2, and p. 1108 for Study 3).

## From TASS to NIPS

We propose that slight modifications to the TASS model can remedy its weaknesses and inconsistencies. Our modifications are fivefold and transform the TASS model into the NIPS model. The first modification deals with Marshall and Brown (2006) choice of nonprovoking situations as representing the lowest level of the situation factor. Cooper and Withey (2009) argue that testing person × situation interactions requires great care in pairing situations and personality constructs (p. 69). More specifically, they request the choice of situations that are functionally equivalent to the personality constructs at hand. In line with this quest for functional equivalence, we argue that choosing a zero level of provocation is disadvantageous because situations containing a zero level of provocation may differ *qualitatively* from situations with moderate to high provocation levels. Thus, nonprovoking situations cannot be mapped onto the same underlying quantitative dimension as situations containing at least some level of provocation. Therefore, we replace the nonprovoking situation by a situation that contains a *low* level of provocation. The second modification changes the labeling of the situational levels. In order to avoid confusion with Mischel (1973) concept of weak vs. strong situations, the situation originally labeled “strong” will be renamed “high.” The third modification addresses the problematic assumption that low trait-aggressive individuals are unaffected by a moderate provocation. Instead, we assume that a moderate provocation level (as compared to a low provocation level) will have at least some effect on all individuals, including those low on trait aggressiveness. The fourth modification is that we allow for nonlinear trait effects. This requires a third level of the trait factor. Consequently, we add a moderate trait level. The fifth modification is that we allow for larger trait effects among low and high situational provocations.

**Figure 2:**
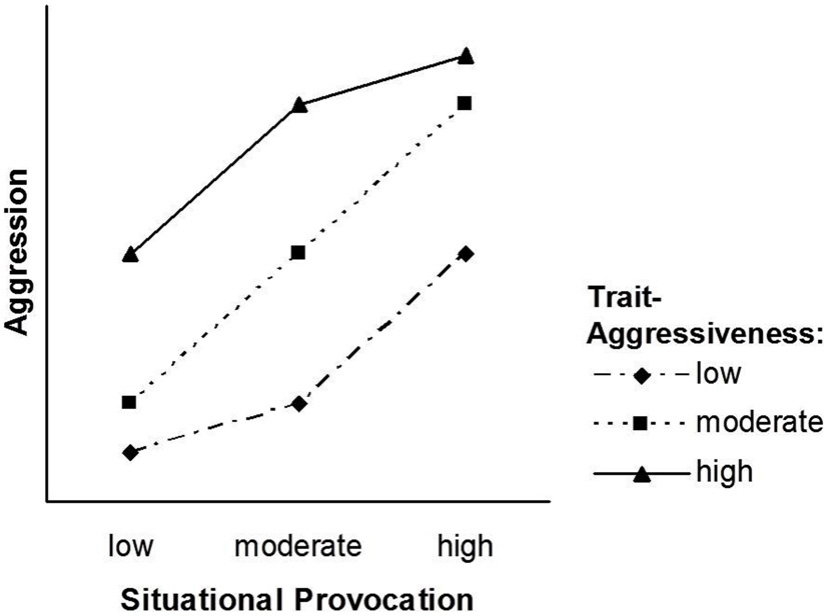
**The NIPS model with three trait levels**.

**Figure 3:**
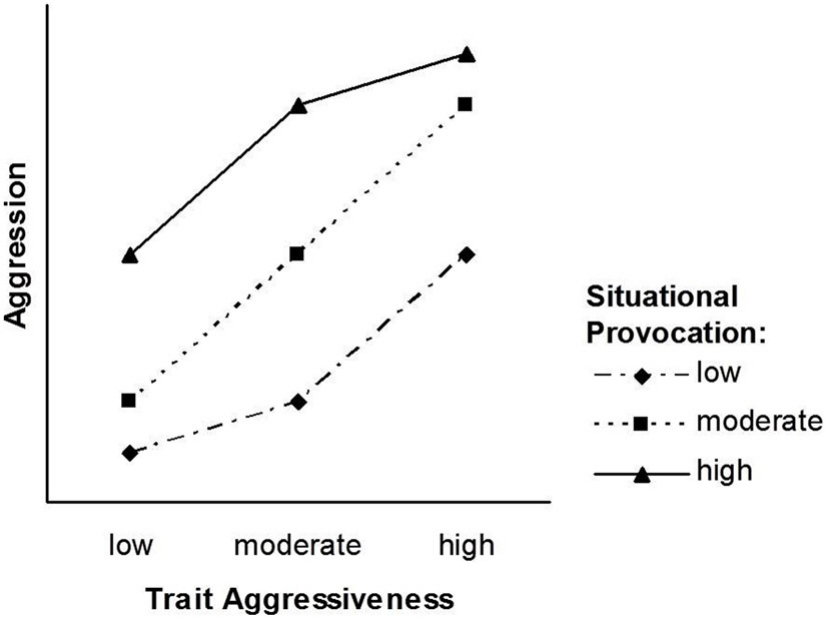
**The NIPS model after exchanging the formal status of situational provocation and trait aggressiveness**.

The pattern that follows from our transformation of the TASS model into the NIPS model is depicted in Figures 2, 3. Figure 2 displays the situation factor on the *X*-axis, whereas the three trait levels are displayed as separate lines. Figure 3 displays the trait factor on the *X*-axis, whereas the three situational levels are displayed as separate lines. Whereas Figure 2 shows that the effect of situational provocation on aggression cannot be generalized across levels of trait aggressiveness, Figure 3 shows that the effect of trait aggressiveness on aggression cannot be generalized across levels of situational provocation.

### Symmetrical interaction patterns

Unlike the TASS model, the NIPS model allows for a symmetrical person × situation interaction. A two-way interaction is symmetrical if both ways of depicting the interaction (one factor on the abscissa and the other as separate lines) yield an identical pattern (cf. Figures 2, 3). Figure 2 is entirely in line with the basic premise of the TASS model: The difference between low and moderate provocations is larger for high trait-aggressive individuals (as compared to low trait-aggressive individuals), whereas the difference between moderate and high provocations is larger for low trait-aggressive individuals (as compared to high trait-aggressive individuals).

Due to the symmetrical nature of the NIPS model, the nonlinearity of the situation effect (Figure 2) is mirrored by the nonlinearity of the trait effect (Figure 3). Nonlinear effects of trait aggressiveness across situations are, as we have argued earlier, conceptually implied by the threshold notion of TASS. Thus, the pattern of the NIPS model is certainly in agreement with Marshall and Brown (2006) core ideas and follows them through to their logical conclusion.

Note that the possibility of a symmetrical person × situation interaction is precluded by the TASS model because such an interaction pattern requires that trait effects at low and high points on the situation dimension are equally as large as situation effects at low and high points on the trait dimension. The TASS model, however, assumes that trait effects are virtually zero at low and high points on the situation dimension. Marshall and Brown (2006) do not offer convincing theoretical reasons for this assumption. Moreover, the asymmetric pattern of the TASS model is not in agreement with a prominent psychometric model, the Rasch model (Rasch, 1960, 1961). As we will show next, this model matches the NIPS model closely but not the TASS model.

## Relating the NIPS model to psychometric theory and psychometric concepts

### Linking the NIPS model to the rasch model

A link between the NIPS model and the Rasch model seems possible because psychometric theories imply models that serve the same purpose that person × situation models are designed for. Just like the TASS model and the NIPS model, psychometric theories describe the functional relation between a behavior and the causes of that behavior. We propose that unlike the TASS model, the NIPS model can be easily linked to the Rasch model. This is true because, unlike the effect pattern of the TASS model, the effect pattern of the NIPS model is highly similar to the characteristic curve of the Rasch model. We claim that this similarity adds to the plausibility of the NIPS model.

**Figure 4:**
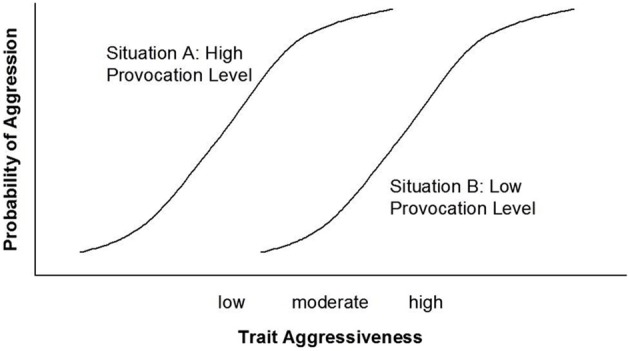
**Item characteristic curves of two situations differing in degree of provocation**.

**Figure 5:**
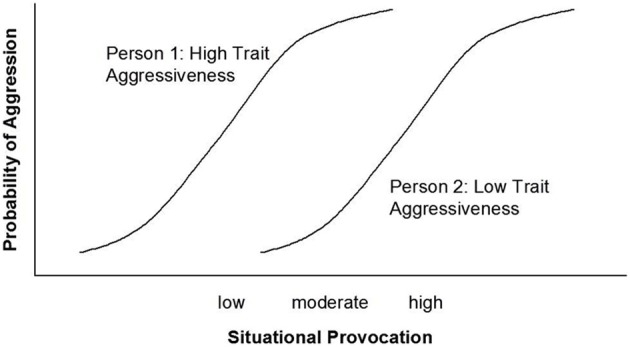
**Person characteristic curves of two individuals differing in trait aggressiveness**.

The Rasch model describes how the probability of solving a task (e.g., an item from an intelligence test) simultaneously depends on the item's difficulty and the person's ability. Item difficulty and person ability are conceived of as continuous variables that can be projected onto the same interval scale. Solving vs. not solving an item is a binary event. According to the Rasch model, such an event cannot linearly depend on item difficulty, nor can it linearly depend on person ability. Item Characteristic Curves (ICC) describe how the probability of solving an item depends on both the item's difficulty and on the ability of the person considered. These concepts—item difficulty and person ability—originate from ability testing. Given their formal definition, however, these concepts can be applied to any psychological domain in addition to ability. The literature contains a large number of successful applications of the Rasch model and extensions of this model to a variety of psychological constructs (e.g., for personality assessment; Reise and Waller, 1990; Chernyshenko et al., 2001). Figure 4 presents the ICCs of two items, Situation A and Situation B, the former having a higher level of provocation than the latter.^2^ Figure 5 depicts Person Characteristic Curves (PCCs) for two people who differ in trait aggressiveness (for further information on the Rasch Model, ICC, and PCC, see, e.g., Lord, 1980; Embretson and Reise, 2000).

**Figure 6:**
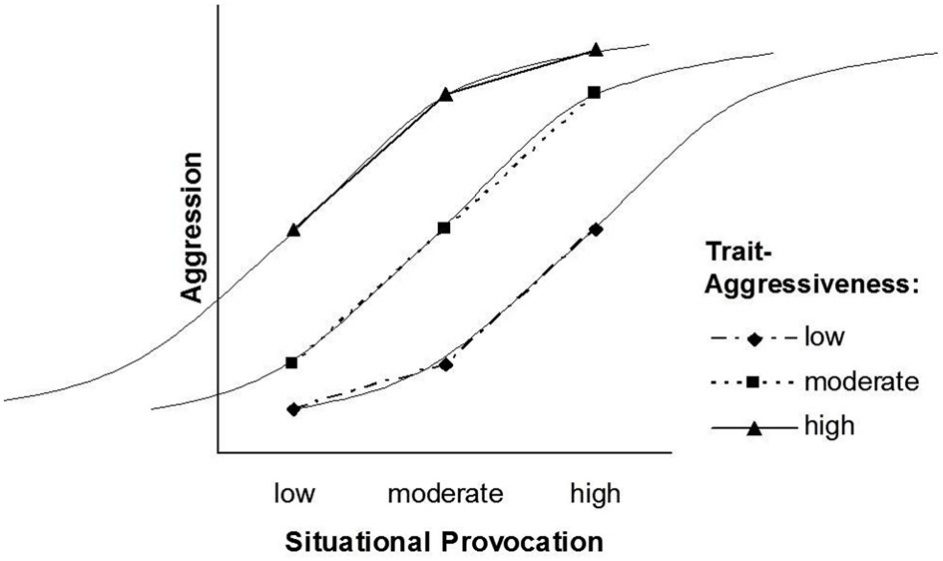
**Integrating person characteristic curves into the NIPS model**.

**Figure 7:**
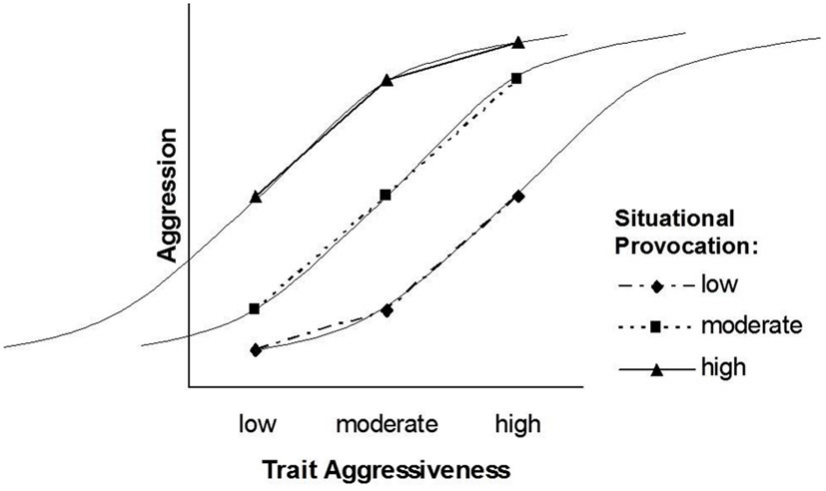
**Integrating situation characteristic curves into the NIPS model**.

Ignoring for the moment that the dependent variables of the NIPS model and the Rasch model have different scales, the former being an intensity scale and the latter being a probability scale, a striking similarity can be observed when comparing Figures 2, 5. In both figures, the difference in the degree or in the likelihood of aggression between a person high in trait aggressiveness and a person low in trait aggressiveness is smaller in situations that have either a low or a high provocation level as compared to moderately provoking situations. The same similarity can be observed when comparing Figures 3, 4. In these two figures, the difference in the degree or in the likelihood of aggression between a situation low in provocation level and a situation high in provocation level is smaller for people who are either high or low in trait aggressiveness as compared to people with a moderate level of trait aggressiveness. These similarities become even more obvious when we integrate the NIPS model and the Rasch model graphically into Figures 6, 7. These figures also demonstrate that the expected levels of aggression for individuals with a moderate trait aggressiveness level and for situations with a moderate provocation level fit the corresponding ogives (i.e., curves) of the Rasch model well.

One might object that the binary behavior scale of the Rasch model (displaying aggression vs. not displaying aggression) and the interval behavior scale of the NIPS model (intensity of aggression) prohibit a direct comparison of the two models. This is true, of course, in a formal sense. However, the basic idea and purpose of the Rasch model may enhance one's understanding of the NIPS pattern, and this is why linking the two models on a conceptual level is fruitful. The Rasch model was proposed to handle the prediction of behavior that is limited in range. The very same reasons (i.e., limits to the range of the intensity or frequency of behavior) may also explain data patterns that are consistent with the NIPS model. In fact, this seems highly likely because most psychological variables are not endless but are instead limited in range. These limits do not need to be artifacts resulting from tailored scales. Rather, they may be due to biological/physiological limits, social limits, or limits imposed by the person via self-control.

Many examples could be given for such limits. In monopolar constructs (e.g., anxiety or aggression), the *lower* bound of the behavior scale is obvious because it is defined by the total absence of the relevant behavior. Moreover, it is also reasonable to assume that both the frequency and the intensity of behaviors have *upper* bounds. If we measure aggression as the intensity of punching an object or another person, there certainly is an upper intensity limit. If we measure spider phobia as the speed at which a person runs away from spiders, there clearly is a biologically determined upper limit to the scale as well.

Upper boundaries of behavior variables do not originate only from biological causes. They might also be invoked by social factors and self-regulation processes. Many extreme behaviors such as aggression meet with social disapproval because they deviate from a social norm. Because people are generally aware of social norms, are able to anticipate the negative consequences of violating these norms, and are motivated to avoid negative consequences, they engage in self-control and self-regulation. Quite often, avoiding negative consequences can be achieved only by behaving within the limits set by a norm.

Of course, these examples do not cover the entire spectrum of human behavior, but they are examples that illustrate the idea that both the intensity and the frequency of behavior often cannot vary endlessly. Whenever this is true, the NIPS model will be superior to models that assume additive linear effects. It is obvious, however, that the NIPS model is a simplification, as every model is. Various extensions and refinements are possible and plausible. It is possible, for example, that not every person will be affected by the limits named above to the same extent. The upper limits could be given by the degree of norm internalization or self-control. This would mean that the thresholds could vary from person to person in a systematic way. We do not want to ignore these potentially interesting mechanisms, but we present the model in its simplest form to avoid making it too complex.

### Weak and strong situations

Marshall and Brown (2006) have linked their model to the concept of *weak* and *strong* situations (e.g., Mischel, 1973; Price and Bouffard, 1974). Strong situations can be defined as situations in which individual differences in behavior are restricted, for instance due to norms, conventions, and rituals. Strong situations trigger uniform behavior. Very few people deviate from the norm. By contrast, weak situations can be defined as situations in which individual differences in behavior are unrestricted because no standardizing norm is salient. Marshall and Brown apply this concept to aggressive behavior as a response to different levels of situational provocations. They implicitly argue that very low and very high provocation levels are special cases of strong situations. This is why they assume that trait effects will be virtually zero in these conditions (see Figure 1).

In psychometric terms, a situation containing low levels of provocation would be a *difficult item* because almost nobody will behave aggressively. A situation containing high levels of provocation would be an *easy item* because many people will react aggressively. Because the interindividual variability of behavior is reduced in strong situations, these situations cannot discriminate between people as effectively as weak situations can. This principle is well known in psychometrics. Very easy and very difficult items are less able to discriminate between individuals than moderately difficult items.

**Figure 8:**
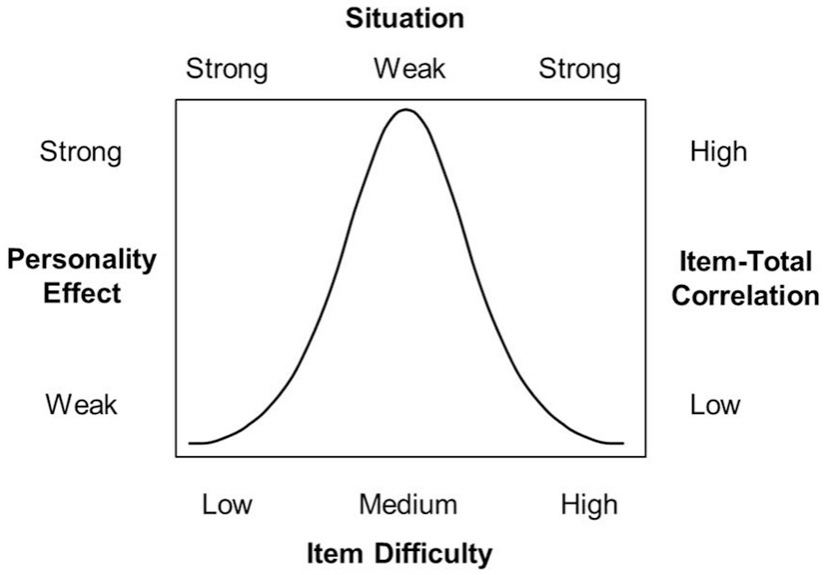
**Links between the concepts of situation strength, item difficulty, personality effect, and item-total correlation**.

Figure 8 integrates the concepts and principles we have discussed. First, Figure 8 shows how weak and strong situations are related to easy and difficult items. Second, Figure 8 shows that difficult and easy items have little discriminative power (low item-total correlations) as compared to moderately difficult items. This again corresponds with Item Response Theory. Each ICC possesses an item information function that has the same shape that we assume for the discriminative power of situations (cf. Lord, 1980). Third, Figure 8 shows that the impact of personality traits on behavior, sometimes called the personality effect, depends monotonically on the strength of the situation. Given the curvilinear relation between the strength of a situation and its difficulty, the impact of personality traits on behavior is also curvilinearly correlated with the difficulty of situations.

### Weak and strong persons

Weak vs. strong situations generate only one among several possible person × situation interaction patterns. If extreme, they may prohibit any trait effect. A provocation may be so strong (psychometrically *easy*) that everybody reacts aggressively to it. Other (psychometrically *difficult*) situations may contain so little provocation that nobody reacts aggressively. Thus, combining extremely strong and extremely weak situations implies that *the situation shapes the magnitude of trait effects.*

**Figure 9:**
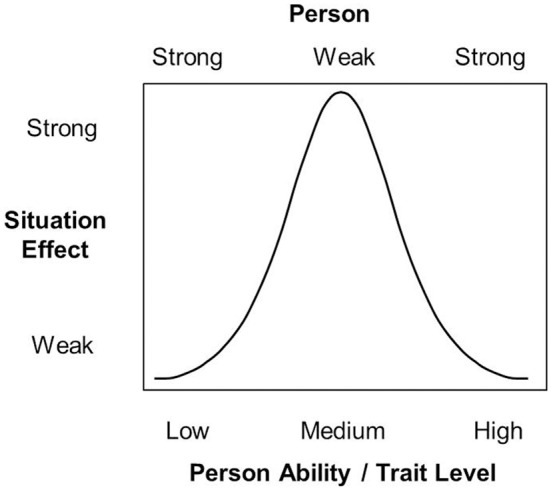
**Links between the concepts of person strength, person ability or trait level, and situation effect**.

Now let us consider a different person × situation interaction model that makes entirely different predictions. In this interaction model, *personality shapes the magnitude of situation effects* such that the possible range of situation effects is determined by the person's trait score (and not vice versa). In psychometric terms, we might speak of *strong* and *weak persons* (instead of situations). *Strong* persons display little intraindividual differences in behavior across situations. Very low and very high trait levels are *special cases* of strong persons.^3^ For example, people who are extremely low in trait-aggressiveness may respond nonaggressively regardless of how much they are provoked. People extremely high in trait-aggressiveness may respond aggressively regardless of how little they are provoked. By contrast, moderately trait-aggressive individuals may show considerable variation in aggressive responding across a range of different provocation levels. Thus, people with moderate trait levels represent *weak* persons. Figure 9 demonstrates how person strength, the trait level, and the situation effect are related to each other.^4^ Note that such a model makes entirely different predictions than the TASS model. The TASS model makes clear assumptions regarding the relative strength of person and situation effects. It assumes that situations shape the magnitude of trait effects, but not vice versa. However, Marshall and Brown (2006) own data are not fully consistent with this assumption.

The NIPS model reconciles the two kinds of person × situation interaction models: It assumes that the range of trait effects is smaller for low and high levels of the situation factor, and it also assumes that the range of situation effects is smaller for high and low levels of the trait factor. The pattern depicted in Figures 2, 3 suggests that the way situations shape the magnitude of trait effects is identical to the way personality shapes the magnitude of situation effects. This symmetry assumption is well in line with the Rasch model, and the Rasch model has received empirical support in many applications.

It is important to understand that the interactive quality of the NIPS model results from the conditional nature of both trait effects and situation effects. All trait effects and all situation effects are conditional effects. The strength of a situation effect (difference in behavior between two situations) always depends on the trait level. Accordingly, the strength of a trait effect (difference in behavior between two persons) always depends of the situation level. Interactions are defined as differences between differences. In the NIPS model, differences between situations differ between persons. Accordingly, differences between persons differ between situations.

## Explanatory capacities of the NIPS model

Just like the TASS model, the NIPS model is a descriptive model. Neither model specifies the psychological mechanisms that cause the patterns of behavior they predict. However, combining these models with substantive theory can bestow explanatory capacity upon them. Marshall and Brown (2006) propose the general threshold concept as an explanation for this. The threshold concept has two limits, however. First, it is not an explanatory concept. Second, it cannot explain why initial differences in behavior between individuals become increasingly larger as we move from difficult to moderately difficult situations. It is also not able to explain why individual differences in behavior become smaller again as we move from moderately difficult situations to easy situations. These properties of the NIPS model would require complementing the threshold concept with the concept of thresholds for the perception of *differences* between situations. However, even adding these thresholds would not satisfy the need for *substantive* explanations. What theories might contain explanations for the NIPS pattern?

Synergy is one concept that might help generate substantive explanations for person × situation interactions (Schmitt et al., 2003). The term synergy, derived from systems theory (Von Bertalanffy, 1968), denotes a conceptual or mathematical product of functionally equivalent factors and is used both in basic (biology, chemistry, physics) and applied sciences (medicine, pharmacy, meteorology) as a general explanation of nonlinear effects and change. In biology, for instance, synergy means that the overall effect (e.g., on the growth of a biological structure) of several factors (e.g., hormones or nutrition) is larger than the sum of their single effects. Factors are considered functionally equivalent if they affect an outcome variable such as behavior or change similarly and are thus exchangeable. Applied to aggressive behavior, trait aggressiveness and situational provocation are functionally equivalent because they both affect aggressive behavior.

In fact, synergistic interactions that account for the diverging part of the slopes of the NIPS model as shown in Figures 2, 3 have been assumed in state-trait anxiety theory and state-trait anger theories as proposed by Endler (1975) and Spielberger (1972). They have also been demonstrated in other psychological domains such as justice judgment and behavior (Schmitt et al., 2003; Schmitt and Sabbagh, 2004). Several psychological principles have been proposed for explaining synergistic interactions, the most influential being schema theory (Markus, 1977). Schemas filter and guide information processing such as attention, information search, memory, and complex inferences like causal attributions. Because the availability and accessibility of schemas vary with underlying personality traits, information is processed in a manner that is congruent with these traits (Rusting, 1998). Persons high in a certain trait detect minimal trait-congruent characteristics in ambiguous situations due to selective attention and selective information search. They give more weight to trait-congruent information in judgment and decision processes than to irrelevant or incongruent information also available in an ambiguous situation. Highly trait-anxious individuals, for instance, selectively attend to threatening information and put more weight on threat signals than on security signals. As a consequence, they are not only alarmed earlier than individuals low in trait anxiety but also react more strongly to situations that differ in threat (Endler, 1975). Highly aggressive individuals, to provide a second example, have schemas that include a hostile attribution bias (Dodge, 1980). They tend to interpret a disturbing but ambiguous event more readily as the consequence of purposeful behavior and thus as a provocation. Consequently, they are prone to respond to slight provocations with counter-aggressive and retaliatory acts. In addition to anxiety and aggression, justice sensitivity can be given as a third and more recent example. Several studies have shown that justice sensitive individuals selectively attend to justice-related cues in a situation of potential injustice and tend to interpret ambiguous situations as just or unjust based on these cues. As a consequence, they react more strongly with justice-related emotion (anger, outrage, guilt) and behavior such as compensating innocent victims and punishing perpetrators (Baumert and Schmitt, 2009; Baumert et al., 2011, 2012).

The mechanisms that turn a synergistic or conjunctive interaction (diverging slopes in Figures 2, 3) into a disjunctive interaction (converging slopes in Figures 2, 3) have been addressed less often in the literature. From the perspective of general system theory (Von Bertalanffy, 1968) and the principle of homeostasis, one might argue that the turn from a synergistic into a disjunctive interaction is inevitable for preventing the system from exploding or falling out of balance. This process seems particularly plausible for systems that would suffer damage from states that exceed the boundaries within which the system functions properly. This reasoning is consistent with our earlier suggestion that biological characteristics and social norms place limits on the frequency and intensity of behavior. Most likely, however, these are not the only mechanisms. The schema concept that has been employed for explaining synergistic interactions may also explain why this interaction turns into a disjunctive interaction at a certain point on the difficulty scale of situations.

The application of schema theory to the disjunctive part of the NIPS interaction seems possible if we make three assumptions. *First*, we assume that a cognitive schema works like a pattern recognition tool: A critical number of elements that define the pattern have to be identified before the Gestalt of the pattern will be recognized. *Second*, we assume that a difficult situation contains fewer or less obvious indications of the elements that define the pattern. Accordingly, easy situations contain more or more obvious indications of critical elements. *Third*, we assume that individuals high on a trait have more sensitive and finely tuned schemas and will, for this reason, more easily recognize and combine the elements of a pattern. Combining all three assumptions implies that the situation schemas of individuals high on the trait will become *saturated* relatively quickly compared to individuals low on the trait. For people low on the trait, more obvious indications or a larger number of them are necessary to activate the schema, and this is more likely to happen in easy situations than in difficult ones. For people high on the trait, additional information contained in easy situations is redundant because their schema can be fully activated with a smaller number and less obvious indications of critical elements. Additional information at a certain point cannot increase the degree of activation of a specific schema. This idea is speculative. However, it is consistent with the NIPS model and adds an explanatory component to this model. Moreover, it is a parsimonious idea because it can explain both parts of the person × situation interaction (i.e., its synergistic or conjunctive part and its disjunctive part).

In fact, preliminary evidence from our justice sensitivity research mentioned earlier is consistent with our reasoning. Yet unpublished data show that priming participants with unfairness can have a weaker effect on justice-related emotion and behavior for highly justice insensitive individuals compared to individuals with low justice sensitivity. This pattern suggests that for highly justice sensitive individuals, schemas of justice-related situations and events are chronically accessible and therefore cannot be made much more accessible by situational cues.

## Directions for future research

More research is needed before the NIPS model can be accepted as a general person × situation interaction model of behavior. Future research has to solve several questions, some of which will be addressed in this last section of our paper.

### How can we measure and define strong and weak persons and situations?

First, future research has to find and systematically compare strategies that can be used to measure the strength of persons and situations (Cooper and Withey, 2009). Assuming that strong persons are those with extremely high or low trait levels, the measurement of person strength seems straightforward at first glance. However, in taking a closer look at the way psychologists measure traits, a problem becomes obvious. The items of many personality questionnaires describe behavior in situations. The item “If somebody hits me, I hit back” from the Buss and Perry (1992) Aggression Questionnaire serves as a good example. Items like these raise several important questions. How can we independently measure the three components of the NIPS: person, situation, and behavior? Will the model become circular if the components are not measured independently? Funder (2006) notion of the personality triad may help answer these questions. He states that each of the triad's components—person, situation, and behavior—has to be understood in terms of the other two. If they are inseparable, each of them has to be measured by taking the other two into account. Does this strategy make the NIPS model circular? We do not believe so because the nonlinear shape of the interrelation is not an implication of their functional interdependence in Funder's personality triad.

Any empirical test of the NIPS model requires at least three levels of the situation factor. If we assume that situation factors are metric in nature, as was assumed in the studies conducted by Marshall and Brown (2006), then a proper scaling of the situation variable is crucial. For example, how can we define low, moderate, and high levels of situational provocation? From the perspective of the personality triad, this is possible in the same way in which we measure trait levels. Trait levels represent averages of behavior across many situations. Accordingly, situation levels can be measured by averaging the behavior of individuals. This is in fact the way item difficulties are defined in psychometrics. Note that both strategies of measuring trait level and situation level do not imply a specific shape of the functional relation between behavior level, trait level, and situation level. Thus, the NIPS model can be tested using the personality triad as defined by Funder (2006) and as employed in recent empirical studies (e.g., Sherman et al., 2010). Nevertheless, we are not saying that aggregating the behavior of people is the only or best way of measuring the situation level. In fact, we have used other strategies in our own research and will describe these briefly in the next section.

### How much empirical support has the NIPS model received so far?

We have started with empirically testing the NIPS model in the domains of aggression, jealousy, and well-being. The results of our first studies are encouraging. In light of the recent debate on the replicability and generalizability of findings (Asendorpf et al., 2013), however, we feel that more research is needed before safe conclusions about the validity of the NIPS model can be drawn. Importantly, additional studies should not come solely from our lab. Rather, it seems crucial for the credibility of findings and validity of the NIPS model that other research groups also submit it to empirical tests. Making this possible was the main reason for our decision to publish the model in a theoretical paper at this point.

Our first studies were aimed at comparing different methods of scaling situations. A first method was inspired by schema theory and consisted of varying the number of relevant hints suggesting that a situation has a certain quality (being provocative) and a certain intensity of that quality (degree of provocativeness). A second strategy employed expert ratings of the quality and intensity (levels) of situations (cf. Wilkowski et al., 2007). This approach corresponds to what is known as stimulus scaling in psychometrics. As a third strategy, we aggregated the behavior of persons in situations according to the personality triad framework. This method corresponds to what is known as response scaling in psychometrics.

These few examples from our ongoing research show that we consider our model to be a general person × situation interaction model that is not limited to a few constructs but rather applicable to a large spectrum of behavioral domains. Nevertheless, person × situation interactions may be more pronounced in some behavioral domains than in others. In other words, the type of construct or behavior may be a moderator that determines the overall strength of person × situation interactions. Identifying these moderators would be a valuable task for future research.

### What are the psychological mechanisms behind the NIPS pattern?

Our brief analysis suggests that although the model might be a general and robust model on the descriptive level, several biological and psychological mechanisms might be responsible for the characteristic pattern. In our justice sensitivity research, we found preliminary evidence for the schema account we suggested earlier as a possible mechanism that can explain both the diverging and the converging parts of the interaction. However, more studies that systematically combine trait levels of justice sensitivity with levels of situational injustice are needed before our first results can be considered to be robust.

Most likely, more mechanisms besides the few we have proposed can explain the NIPS pattern. We would like to encourage other researchers to link the NIPS model with the psychological mechanisms they are studying, and we are looking forward to seeing if parts of their data fit with the NIPS model. This leads us to the last question we want to address.

### How can the NIPS model be used in future research?

We suggest and hope that the NIPS model will provide a common framework for scholars who conduct research in social psychology and personality psychology. Because the model is a general model that can be applied in principle to a wide range of traits, situations, and behavioral domains, the model may provide a simple but common language for personality researchers and social psychologists alike. The concepts of strong vs. weak persons and situations as well as the nonlinear interaction pattern of the model may facilitate communication among scholars from different research areas. The model may also contribute to the comparison of results from different studies and serve as a common framework for meta-analyses on person × situation interactions.

Next, the NIPS model may help to resolve inconsistencies among findings from different studies. According to the model, inconsistent findings may result from a limited range of trait levels and situation levels. Depending on the range of trait levels and the range of situation levels that were combined in a study, either conjunctive or disjunctive interactions may result if the model fits the data. Without considering the full pattern of the NIPS model, such findings could easily be mistaken as inconsistent or even contradictory. However, they might be fully consistent with the NIPS model and therefore also consistent among each other as well. Reanalyses of data that were considered to be inconsistent up to now may show that the data are in line with the NIPS model. In this case, these data would no longer appear to be inconsistent but rather will be viewed as coherent and conclusive.

The NIPS model can also be used to clarify the polarity of traits. The data pattern presented in this paper is the one to be expected for unipolar constructs. Bipolar constructs should reveal the same data pattern twice (with one part being the mirror inversion of the other). Testing the unipolar version of the model against the bipolar version in a specific application can thus contribute to the empirical clarification of the polarity of a construct.

We hope that these examples will encourage readers to consider the application of the NIPS model to their own research questions and data. For the sake of replication, we look forward to applications of the NIPS model to the constructs of our own work in progress (aggression, jealousy, well-being, justice). We also hope for applications to other behavioral domains because only a substantive diversity of applications can shed light on the generalizability of the NIPS model.

### Conflict of interest statement

The authors declare that the research was conducted in the absence of any commercial or financial relationships that could be construed as a potential conflict of interest.

## Footnotes

^1^Note that Figure 1 and the remaining figures represent relations between independent and dependent variables schematically. Therefore, the figures do not contain scale units. The scales may differ between substantive applications of the model and between measures of the variables to which the model is applied. Therefore, the values labeled “low,” “moderate,” and “high” do not represent well-defined points on a scale. Rather, they indicate ranges on the scale that measure the constructs according to Marshall and Brown (2006) or constructs of other applications of the TASS model. Only the ordinates of Figures 4, 5 are well-defined. They represent the probability scale that ranges from 0 to 1.

^2^On ability tests, easy items are items that have a higher probability of endorsement, even by people with low ability, and are thus located on the left side of the ability scale. Items that are more difficult have a low probability of being endorsed until a person has a higher ability level, and thus, these items are located further to the right on the ability scale. Transferring this idea to the domain of aggression explains why “Situation A: High provocation level” is located on the left side of the scale—because it does not take much trait aggressiveness (or “ability”) in this situation to act aggressively with some probability. Likewise, “Situation B: Low provocation level” is analogous to the difficult item, and is thus located on the right side of the scale where it takes a higher level of trait aggressiveness to have some probability of responding in this situation.

^3^Note that extreme trait levels are not the only possible reason for why a person's behavioral variability will be smaller than the behavioral variability of other individuals. People may also display little trans-situational variability in behavior due to other factors such as self-monitoring (Snyder and Tanke, 1976; Snyder, 1987), self-consciousness (Fenigstein et al., 1975), need for closure (Webster and Kruglanski, 1994), preference for consistency or other metatraits (Bem and Allen, 1974; Baumeister and Tice, 1988), and moderators of consistency that have been identified (Snyder and Ickes, 1985). Individuals with high scores on these traits are also strong persons, although their scores on the target trait might not be extreme. Nevertheless, there is clear empirical evidence that shows that trait extremity is inversely related to cross-situational variability (Jackson and Paunonen, 1985; Baumeister and Tice, 1988; Paunonen, 1988). Therefore, it seems safe to say that people with very low and very high trait scores are strong persons, although individuals may differ in cross-situational variability in behavior for additional reasons.

^4^Trait-aggressiveness does not appear to fit neatly into the “weak” vs. “strong” situation framework. In a recent meta-analysis, Bettencourt et al. (2006) showed that trait-aggressiveness actually predicts aggressive behavior regardless of the level of situational provocation. In other words, trait-aggressiveness yields main effects, but interactions with situational provocations are much less frequent. Other traits such as impulsivity, emotional susceptibility, or narcissism yielded much larger interactions with situational variations.
